# Impact of Religious Affiliation on Clinical Outcomes in Liver Transplant Patients

**DOI:** 10.7759/cureus.66372

**Published:** 2024-08-07

**Authors:** Cara C Prier, Mary S Hedges, Leila M Tolaymat, Ashley L Walker, Claire Haga, Emily C Craver, Michael G Heckman, Mingyuan Yin, Mindy McManus, Nancy Dawson, Andrew P Keaveny

**Affiliations:** 1 Internal Medicine, Mayo Clinic, Jacksonville, USA; 2 Dermatology, Mayo Clinic, Jacksonville, USA; 3 Family Medicine, Mayo Clinic, Jacksonville, USA; 4 Biostatistics, Mayo Clinic, Jacksonville, USA; 5 Research Administration, Mayo Clinic, Jacksonville, USA; 6 Coaching, Mindy's Executive Coaching, Jacksonville, USA; 7 Gastroenterology and Hepatology, Mayo Clinic, Jacksonville, USA

**Keywords:** post-liver transplant, short-term transplant outcomes, transplant survival, religion and medicine, spirituality and medicine

## Abstract

While the impact of spirituality as it relates to quality of life post-liver transplant (LT) has been studied, there are limited data showing how religious affiliation impacts objective measures such as survival. The aim of the study is to investigate whether LT recipients who identified as having a religious affiliation had better clinical outcomes when compared to LT recipients who did not.

Religious affiliation is obtained as part of general demographic information for patients within our institution (options of "choose not to disclose" and "no religious affiliation" are available). Subjects in this retrospective cohort study which conformed with the Declarations of Helsinki and Istanbul were separated into cohorts: LT recipients who self-reported religious affiliation and LT recipients who did not. All LT recipients between March 2007 and September 2018 who had available information regarding their reported religion were included. Excluded patients included those who received a multi-organ transplant, underwent re-transplantation, received a partial liver graft, and identified as agnostic. Outcomes included 30-day readmission, death, and the composite outcome of re-transplantation/death.

In an unadjusted analysis of 378 patients, there were no statistically significant differences between the two groups for 30-day readmission (OR=1.15, P=0.71), death (HR=0.63, P=0.19), or re-transplantation/death (HR=0.90, P=0.75). In multivariable analysis, adjusting for age at transplant and hospital admittance status when called for transplant, results were similar.

We found no statistically significant difference in the outcomes measured between patients with and without self-reported religious affiliation.

Further studies into the role of participation in religious activity and the impact of engagement with a religious community should be conducted in the future.

## Introduction

The study of spirituality and its impact on quality of life has resulted in several publications in recent years [1-4]. Prior research supports the beneficial impact of having a religion on patients' physical and mental health outcomes [5]. Patients with religious involvement were reported to have better cardiac surgery outcomes, greater longevity, and a lower rate of cancer attributed to healthier lifestyles such as avoidance of smoking and alcohol use [5]. Patients with religious involvement also experienced more positive emotions and fewer mental health disorders (specifically depression, anxiety disorder, substance abuse, and suicide) attributed to community involvement, social support, and stress reduction [5]. 

Many factors affect long-term health outcomes in liver transplant (LT) recipients including the etiology of liver disease, other medical comorbidities, as well as complications related to the surgery itself and to immunosuppressive therapy [6]. The concept of survivorship care has been well established in cancer treatment to improve long-term outcomes as it focuses on patients' health and well-being from the time of diagnosis through treatment and beyond. This model has been recommended as a holistic approach to patient care as it focuses on an array of dimensions including physical, social, psychological, and spiritual well-being [7]. This is supported by studies which show that religiosity has been associated with improved quality of life and well-being in solid organ transplant recipients [8-11]. Spiritual well-being is also associated with increased compliance with immunosuppressive medications in LT recipients [12]. One study in 2010 of LT recipients demonstrated that religiosity (specifically "searching for God") was associated with prolonged survival [13]. To our knowledge, no other studies have assessed whether religious affiliation is associated with improved overall health outcomes in transplant patients, specifically the measurable outcomes of survival, need for re-transplantation, and hospital readmission. 

The aim of this study was to assess clinical outcomes in two cohorts of LT patients: those who have a religious affiliation and those who did not have a religious affiliation. We hypothesized that LT recipients who reported a religious affiliation would have decreased hospitalizations, decreased rates of re-transplantation, and improved overall survival compared to those recipients without a reported religious affiliation.

## Materials and methods

Study subjects

This was a retrospective cohort study approved by the Institutional Review Board of Mayo Clinic (approval number: 22-006393) declared to be minimal risk. It received a waiver of informed consent and, as such, no patient consent was required. The study conformed with the Declarations of Helsinki and Istanbul. This study was conducted in an academic tertiary care center. All LT recipients between March 2007 and September 2018 who had available information regarding their reported religion were included. Patients who received a multi-organ transplant, who underwent re-transplantation, who received a partial liver graft, or whose religion was agnostic were excluded. In addition to religious affiliation, baseline recipient characteristics (age at transplant, sex, race, ethnicity, body mass index (BMI), Model for End-Stage Liver Disease (MELD) score at transplant, and cause of liver disease), transplant episode data (hospital admittance status when called for transplant), donor information (donor age, donor gender, donor type, donation after cardiac death or brain death, cold ischemia time), and outcomes data (30-day readmission, death, and the composite outcome of re-transplantation or death) were collected. All patients undergoing evaluation for LT at our center were evaluated by a licensed social worker with extensive experience in transplantation. Patients who were identified to have substance use disorders or psychiatric or behavioral issues were assessed by one of the several psychiatrists assigned to the LT team.

Data analysis

Continuous variables were summarized with the sample median and range. Categorical variables were summarized with the number and percentage of patients. Comparisons of baseline, operative, and donor characteristics between patients with religious affiliation and no religious affiliation patients were made using a Wilcoxon rank-sum test (continuous variables) or Fisher's exact test (categorical variables). Outcomes were compared between the religious affiliation and no religious affiliation groups using unadjusted and multivariable regression models that were appropriate for the nature of the given outcome variables. Specifically, the binary outcome of 30-day readmission was compared between the two groups using logistic regression models; odds ratios (ORs) and 95% confidence intervals (CIs) were estimated for and are interpreted as the multiplicative increase in the odds of 30-day readmission for the religious affiliation group compared to the no religious affiliation group. The time-to-event outcomes of death and the composite outcome of re-transplantation or death were compared between the two groups using Cox proportional hazards regression models; hazard ratios (HRs) and 95% CIs were estimated. Multivariable models were adjusted for any baseline, operative, or donor variable that differed between the two groups with a p-value <0.20. The cumulative incidences of time-to-event outcomes (death and re-transplantation or death) after transplant were estimated using the Kaplan-Meier method, where censoring occurred at the last follow-up date. P-values less than 0.05 were considered as statistically significant, and all statistical tests were two-sided. Statistical analysis was performed using R Statistical Software (Version 4.1.2; R Foundation for Statistical Computing, Vienna, Austria).

## Results

A total of 378 patients were included in our study, of whom 330 (87.3%) had a religious affiliation and 48 (12.7%) had no religious affiliation. A summary of the specific religions in the patients with a religious affiliation is provided in Table 1.

**Table 1:** Summary of religions in patients with a religious affiliation

Religion	No. (%) of patients
American Baptist	1 (0.3%)
Anglican	1 (0.3%)
Assembly of God	5 (1.5%)
Baptist	72 (21.8%)
Baptist General Conference	1 (0.3%)
Buddhist	1 (0.3%)
Charismatic	2 (0.6%)
Christian Church, Church of Christ	14 (4.2%)
Christian Reformed Church	1 (0.3%)
Church of Christ A Cappella	1 (0.3%)
Church of God	3 (0.9%)
Congregational	1 (0.3%)
Coptic Orthodox Church	1 (0.3%)
Disciples of Christ, Christian Church	1 (0.3%)
Episcopal	3 (0.9%)
Evangelical Free	1 (0.3%)
Hindu	2 (0.6%)
Holiness	2 (0.6%)
Islam	3 (0.9%)
Jehovah's Witness	1 (0.3%)
Jewish Faith	6 (1.8%)
Jewish Religion Reformed	1 (0.3%)
Lutheran	8 (2.4%)
Mennonite	1 (0.3%)
Methodist	21 (6.4%)
Non-Denominational	10 (3.0%)
Non-specific Christian	32 (9.7%)
Pentecostal	5 (1.5%)
Presbyterian	7 (2.1%)
Presbyterian USA	1 (0.3%)
Protestant	9 (2.7%)
Roman Catholic	93 (28.2%)
Seventh-day Adventist	3 (0.9%)
Southern Baptist Convention	6 (1.8%)
Spiritualism	1 (0.3%)
The Church of Jesus Christ of Latter-day Saints	3 (0.9%)
Unitarian Universalist	2 (0.6%)
United Methodist Church	4 (1.2%)

A number of variables were compared between the two cohorts, including age at transplant, sex, race/ethnicity, MELD score at transplant, BMI at transplant, and cause of liver disease. Patient characteristics are shown in Table 2.

**Table 2:** Comparisons of characteristics between LT patients who have religious affiliation and LT patients with no religious affiliation The sample median (minimum, maximum) is given for continuous variables. P-values result from Fisher's exact test (categorical variables) or a Wilcoxon rank-sum test (continuous variables). BMI: body mass index; MELD: Model for End-Stage Liver Disease; NASH: nonalcoholic steatohepatitis; NAFLD: nonalcoholic fatty liver; PBC: primary biliary cholangitis; PSC: primary sclerosing cholangitis; ICU: intensive care unit; LT: liver transplant

Variable	N	Patients reporting religious affiliation (N=330)	N	Patients reporting no religious affiliation (N=48)	P-value
Baseline characteristics					
Age at transplant (years)	330	59 (22, 70)	48	56 (19, 70)	0.17
Sex (male)	330	206 (62.4%)	48	33 (68.8%)	0.43
Race	323		48		0.67
White		289 (89.5%)		42 (87.5%)	
Black		16 (5.0%)		2 (4.2%)	
Asian		8 (2.5%)		1 (2.1%)	
Others		10 (3.1%)		3 (6.2%)	
Ethnicity (non-Hispanic)	320	295 (92.2%)	48	46 (95.8%)	0.55
BMI at transplant	330	27.7 (13.7, 48.3)	48	27.9 (16.6, 53.8)	0.44
MELD score at transplant	330	19 (6, 51)	48	17 (6, 43)	0.49
Cause of liver disease	330		48		0.41
Cirrhosis associated with alcohol		46 (13.9%)		9 (18.8%)	
Cirrhosis due to NASH/NAFLD/cryptogenic		91 (27.6%)		10 (20.8%)	
Cirrhosis due to viral hepatitis		47 (14.2%)		8 (16.7%)	
Cirrhosis due to autoimmune hepatitis/PBC		17 (5.2%)		6 (12.5%)	
Cirrhosis due to metabolic liver disease		15 (4.5%)		2 (4.2%)	
Hepatocellular carcinoma		70 (21.2%)		9 (18.8%)	
Acute liver failure		3 (0.9%)		1 (2.1%)	
PSC		24 (7.3%)		1 (2.1%)	
Others		17 (5.2%)		2 (4.2%)	
Operative characteristics					
Hospital admittance status when called for transplant	330		48		0.040
Not hospitalized		264 (80.0%)		43 (89.6%)	
ICU		33 (10.0%)		5 (10.4%)	
Hospitalized (non-ICU)		33 (10.0%)		0 (0.0%)	
Cold ischemia time	330	5.9 (2.5, 29.0)	48	5.7 (3.1, 11.3)	0.32
Donor characteristics					
Donor age	330	47.0 (6.0, 81.0)	48	48.0 (13.0, 77.0)	0.35
Donor sex (male)	330	196 (59.4%)	48	27 (56.2%)	0.75
Donation after cardiac death	330	55 (16.7%)	48	11 (22.9%)	0.31

There was a significant difference in hospital admittance status when called for transplant between the two groups (not hospitalized: 80% vs. 89.6%; ICU: 10% vs. 10.4%; hospitalized: 10% vs. 0%; P=0.040). Additionally, though not statistically significant, compared to the no religious affiliation patients, the religiously affiliated patients were slightly older (median: 59 vs. 56 years; P=0.17).

Comparisons of outcomes between the religious affiliation and no religious affiliation groups are shown in Table 3.

**Table 3:** Comparisons of outcomes between LT patients with religious affiliation and LT patients with no religious affiliation Odds ratios, 95% CIs, and p-values result from logistic regression models. Hazard ratios, 95% CIs, and p-values result from Cox proportional hazards regression models. Multivariable models were adjusted for any baseline, operative, or donor variable that differed between the two groups with a p-value <0.20 (age at transplant and hospital admittance status when called for transplant). CI: confidence interval; LT: liver transplant

				Comparison between religious affiliation and no religious affiliation (reference) groups			
	No. (%) of patients			Unadjusted analysis		Multivariable analysis	
Outcome	Has religious affiliation (N=330)	No religious affiliation (N=48)	Association measure	Estimate (95% CI)	P-value	Estimate (95% CI)	P-value
30-day readmission	84 (25.5%)	11 (22.9%)	Odds ratio	1.15 (0.56, 2.35)	0.71	1.30 (0.63, 2.71)	0.48
Death	43 (13.0%)	10 (20.8%)	Hazard ratio	0.63 (0.32, 1.25)	0.19	0.59 (0.29, 1.19)	0.14
Re-transplantation or death	61 (18.5%)	10 (20.8%)	Hazard ratio	0.90 (0.46, 1.75)	0.75	0.90 (0.46, 1.78)	0.77

**Figure 1:**
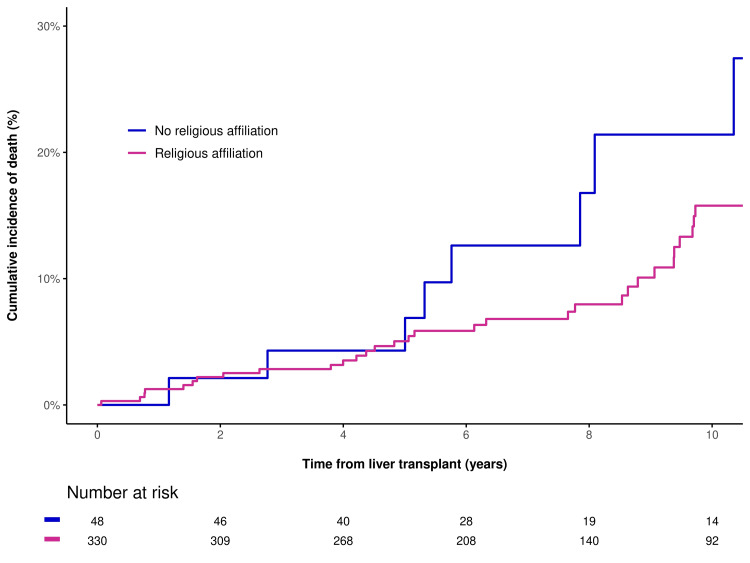
Cumulative incidence of death after LT for patients with religious affiliation and patients with no religious affiliation LT: liver transplant

**Figure 2:**
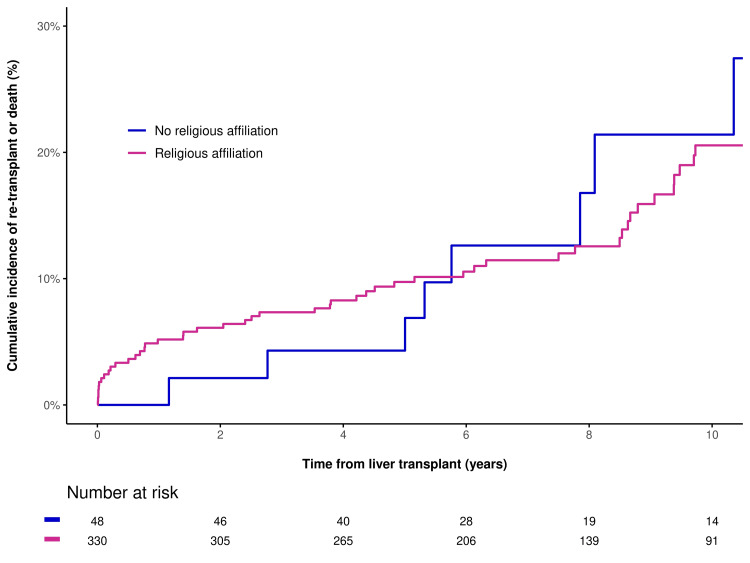
Cumulative incidence of re-transplantation or death after LT for patients with religious affiliation and patients with no religious affiliation LT: liver transplant

In unadjusted analysis, there were no statistically significant differences between the two groups for 30-day readmission (OR=1.15, P=0.71), death (HR=0.63, P=0.19), or re-transplantation/death (HR=0.90, P=0.75). In multivariable analysis, adjusting for age at transplant and hospital admittance status when called for transplant, results were similar for 30-day readmission (OR=1.30, P=0.48), death (HR=0.59, P=0.14), and re-transplantation or death (HR=0.90, P=0.77). This is shown in Figure 1 and Figure 2.

## Discussion

Prior studies have suggested positive health outcomes for patients with religious affiliations including improved quality of life and well-being [8-11]. However, our study of LT recipients did not find a statistically significant difference between LT patients with religious affiliation and those without, using the measured clinical outcomes of survival/death, 30-day hospital readmission, and need for re-transplantation. This finding held true when adjusting for age and hospital admission status.

A negative study can influence practice, removing barriers to transplant that may not be relevant. Our study brings into question whether any emphasis should be placed on religious affiliation prior to transplant, as there does not appear to be predictive value when assessing how patients will fare overall during and after the transplant process. Religious affiliation is not considered in the selection of patients for LT listing at our center, other than if it may impact management (such as accepting blood transfusions). It has been shown that having optimal social support systems in place prior to LT results in an improvement in clinical outcomes, specifically survival [14]. For this reason, our center utilizes other scoring systems such as the Stanford Integrated Psychosocial Assessment for Transplant (SIPAT). This assessment is used in many centers prior to transplant to aid in transplant waitlist decision-making. Patients with higher SIPAT scores have been associated with not being added to the transplant waitlist and have also been found to have an increased risk of immunosuppression non-adherence [15]. While many might consider social support to encompass religion, few studies have specifically addressed the impact of religious affiliation on objective clinical outcomes post-transplant.

Using a questionnaire, Bonaguidi et al. found that patients who identified as believing in God (i.e., trusting in God, having faith in God, seeking God's help, trying to perceive God's will in disease) had prolonged survival post-LT [13]. Our study, however, focused on actual religious affiliation, not overall belief in God.

Strengths

Strengths of our study include a cohort of LT recipients from a high-volume center who all underwent a standardized medical evaluation process, including a comprehensive biopsychosocial evaluation by licensed social workers. There was sufficient time to assess outcomes ranging from four to 15 years post-transplant. Our electronic health record contained discrete fields of patient-reported religious affiliation.

Limitations

Several limitations are important to acknowledge. The sample size was relatively small, particularly that of the group of patients with no religious affiliation, and therefore, the possibility of a type II error (i.e., a false-negative finding) is important to consider. It is possible that a true difference in outcomes exists between the religious affiliation and no religious affiliation groups but that our study did not have sufficient power to detect it. From the electronic records, we were not able to determine whether our patients were actively engaged in religious practice. Furthermore, 88% of the study cohort was Caucasian, the majority of patients lived in the southeastern part of the United States, and Roman Catholicism along with the Baptist faith were the two most commonly identified religious affiliations. Our findings may not be generalizable to other locations, regions, or institutions, where different ethnicities and religious affiliations may be present.

## Conclusions

While many studies have demonstrated the impact of religion and spirituality on well-being and overall quality of life post-LT, few have demonstrated whether or not religious affiliation impacts objective measures such as survival and hospital readmission. While our study did not find a statistically significant difference in the measured outcomes of death, 30-day hospital readmission, and the composite outcome of re-transplantation/death, the results obtained may be useful in determining if religious affiliation should be considered as a criterion to predict how patients will fare overall during and after the LT process. While data suggests that religion and spirituality may improve patients' well-being during the process, our findings do not suggest that it will impact the outcomes measured in this study. The influence of active participation in religious activity and the impact of engagement with a religious community on clinical outcomes post-LT require further study.
